# Genomes of Anguillid Herpesvirus 1 Strains Reveal Evolutionary Disparities and Low Genetic Diversity in the Genus *Cyprinivirus*

**DOI:** 10.3390/microorganisms9050998

**Published:** 2021-05-05

**Authors:** Owen Donohoe, Haiyan Zhang, Natacha Delrez, Yuan Gao, Nicolás M. Suárez, Andrew J. Davison, Alain Vanderplasschen

**Affiliations:** 1 Immunology-Vaccinology, Department of Infectious and Parasitic Diseases, Fundamental and Applied Research for Animals & Health (FARAH), Faculty of Veterinary Medicine, University of Liège, B-4000 Liège, Belgium; owen.donohoe@uliege.be (O.D.); haiyan.zhang@doct.uliege.be (H.Z.); Natacha.Delrez@uliege.be (N.D.); yuan.gao@uliege.be (Y.G.); 2 Bioscience Research Institute, Athlone Institute of Technology, Athlone, Co. N37 HD68 Westmeath, Ireland; 3 MRC-Centre for Virus Research, University of Glasgow, Glasgow G61 1QH, UK; nicolas.suarez@glasgow.ac.uk (N.M.S.); andrew.davison@glasgow.ac.uk (A.J.D.)

**Keywords:** Anguillid herpesvirus 1, cyprinivirus, *Cyprinivirus*, *Alloherpesviridae*, *Herpesvirales*, herpesvirus evolution, positive selection

## Abstract

Anguillid herpesvirus 1 (AngHV-1) is a pathogen of eels and a member of the genus *Cyprinivirus* in the family *Alloherpesviridae*. We have compared the biological and genomic features of different AngHV-1 strains, focusing on their growth kinetics in vitro and genetic content, diversity, and recombination. Comparisons based on three core genes conserved among alloherpesviruses revealed that AngHV-1 exhibits a slower rate of change and less positive selection than other cypriniviruses. We propose that this may be linked to major differences in host species and corresponding epidemiological circumstances. Efforts to derive evolutionary rate estimates for cypriniviruses under various theoretical models were ultimately unrewarding. We highlight the potential value of future collaborative efforts towards generating short-term evolutionary rate estimates based on known sequence sampling dates. Finally, we revealed that there is significantly less genetic diversity in core gene sequences within cyprinivirus species clades compared to species in the family *Herpesviridae*. This suggests that cyprinivirus species may have undergone much more vigorous purifying selection post species clade divergence. We discuss whether this may be linked to biological and anthropogenic factors or to sampling bias, and we propose that the comparison of short-term evolutionary rates between species may provide further insights into these differences.

## 1. Introduction

The order *Herpesvirales* is composed of the three families *Herpesviridae*, *Malacoherpesviridae* and *Alloherpesviridae* [1,2,3,4]. The family *Herpesviridae* consists of herpesviruses of amniotes (reptiles, birds and mammals) and encompasses over 100 recognized species. The family *Malacoherpesviridae* consists of herpesviruses of molluscs and has only two species. The family *Alloherpesviridae* consists of herpesviruses of amphibians (genus *Batrachovirus*) and fish (genera *Salmonivirus, Ictalurivirus* and *Cypriniviru*s) and contains 13 species [5]. Most alloherpesviruses described to date have been recognized because they cause disease outbreaks associated with mass mortalities that have serious impacts on wildlife ecology or the productivity of the aquaculture sector.

All members of the order *Herpesvirales* have a virion that is conserved in structure and contains a large, linear double-stranded DNA (dsDNA) genome. However, the tenuous genetic relationships among the three families indicate that the origin of the order is very ancient [1,2,3,4,5,6]. The most convincingly conserved gene indicating this origin encodes an ATPase involved in viral genome encapsidation that is known as DNA packaging terminase subunit 1 (hereafter called terminase) [1]. In contrast, the common ancestry of species within families is well supported by the existence of conserved (core) genes, with 43 and 12 ancestral genes recognized in the families *Herpesviridae* [7,8] and *Alloherpesviridae* [9], respectively. Some of the core genes in these two families may have the same functions [9]. Prime examples of such genes include genes described as encoding the DNA polymerase catalytic subunit (hereafter called DNA polymerase), the helicase–primase helicase subunit (hereafter called helicase) and the terminase. These also represent some of the most conserved *Alloherpesviridae* core genes among members of the *Cypriniviru*s genus [9,10] and were the main focus of the present study.

The genus *Cyprinivirus* comprises the alloherpesviruses of cyprinids (cyprinid herpesvirus 1, 2 and 3 (CyHV-1, -2 and -3)) and eel (anguillid herpesvirus 1 (AngHV-1)) [5]. CyHV-1 and CyHV-3 infect common and koi carp (*Cyprinus carpio* species), CyHV-2 infects goldfish, Crucian carp and Gibel carp (all of which are *Carassius* species), and AngHV-1 infects Japanese eels (*Anguilla japonica*) and European eels (*Anguilla anguilla*) [9,10]. AngHV-1 causes a hemorrhagic disease in both wild and farmed populations [11] and has mortality rates as high as 30% [12,13,14,15]. This virus has been suggested as one of the causes of the decline of the European eel, which is now classified as a critically endangered species [16]. Notably, unlike cyprinids, European and Japanese eels are catadromous fish. Their lifecycle begins in the open ocean, with larvae drifting on oceanic currents to continental coastal waters, followed by inland migration towards freshwater habitats, where they remain until returning to the sea to spawn before dying [17,18,19]. This complex lifecycle may have had profound effects on the evolution of AngHV-1 in comparison with other cypriniviruses. Since the first description of AngHV-1 in 1986 [20], only two strains have been fully sequenced [10,21]. As a result, insufficient information is available to determine the extent of genetic diversity of this virus.

In the present study, we sequenced seven additional AngHV-1 strains from various geographical origins and compared their growth properties in vitro. Two main genetic lineages were revealed with no apparent correlation to geographical origin. Potential recombination events between some lineages were also detected, and the existence of a third, as yet unidentified, lineage was suggested. The presence of AngHV-1 genes that are disrupted in some strains led to the identification of several genes that are non-essential for growth in vitro. Further analysis of three core genes revealed potential differences in evolutionary attributes between AngHV-1 and other cypriniviruses, and the feasibility of using node calibration to derive absolute estimates of cyprinivirus evolutionary rates was explored. Further comparison of these three core gene sequences and that of a fourth core gene encoding uracil DNA glycosylase (the latter being less well conserved between cypriniviruses), indicated that species clades within the genus *Cyprinivirus* are less diverse than most well-characterized members of the family *Herpesviridae*.

## 2. Materials and Methods

### 2.1. Cells and AngHV-1 Strains

Eel kidney (EK-1) cells [22] were cultured as described previously [23]. The AngHV-1 isolates used in this study are listed in Table 1 together with two fully sequenced isolates that were described previously. AngHV-1 strains DK1, DK2, DK3 and DK4 were kindly provided by Dr Niels Jørgen Olesen and Dr Niccoló Vendramin (DTU Aqua, National Institute of Aquatic Resources, Lyngby, Denmark), strains HVA and CVI by Dr Olga L.M. Haenen (Wageningen University, Wageningen, The Netherlands), and strain UK by Dr Keith Way (Centre for Environment, Fisheries, and Aquaculture Science, Lowestoft, UK). Each strain had been passaged several times before receipt, and all were passaged twice more after receipt.

### 2.2. AngHV-1 Genome Sequencing

Sequencing was performed on the Illumina platform as described previously [25,26]. Approximately 1 million 250 nucleotide paired-end reads were obtained per sample. The annotated sequences were deposited in GenBank (Table 1).

### 2.3. Phylogenetic Analysis of AngHV-1 Genome Sequences

Multiple alignments of genome sequences (Table 1) were made using MAFFT online version 7 (https://mafft.cbrc.jp/alignment/server/, accessed on 3 May 2021) [27]. The alignments were imported into MEGA (v10.1.7) [28], saved as .meg files, reopened again in MEGA and used to identify the best-fitting substitution models based on the lowest Bayesian information criteria (BIC) values. Trees were generated using the unweighted pair group method with arithmetic mean (UPGMA) with a partial deletion of gaps (95% cut-off) and 1000 rounds of bootstrapping were then generated using the best-fitting models from the options available in MEGA. The tree was exported from MEGA in the Newick format (including branch lengths and bootstrap information) and formatted in FigTree (v1.4.4).

### 2.4. Recombination Analysis

Detection of inter-strain recombination, identification of the closest parental sequences, and localization of possible recombination points were carried out using Recombination Detection Program 4 (RDP4) [29], a program that combines a variety of independent methods, including RDP [30], GENECONV [31], BOOTSCAN [32], MaxChi [30], CHIMAERA [33], SISCAN [34] and 3SEQ [35]. Statistical analyses of recombination events were also generated using these methods and implemented in RDP4.

### 2.5. Viral Growth Assay

Triplicate cultures of EK-1 cells were infected with AngHV-1 at a multiplicity of infection (moi) of 0.01 plaque-forming units (pfu)/cell. After an incubation period of 2 h, the cells were washed with phosphate-buffered saline and overlaid with culture medium. The infected cells were scraped off and collected with the supernatant at successive intervals. After centrifugation at 900 × *g* for 30 min at 4 °C, the supernatant was collected and stored at −80 °C. Viral titration was carried out by triplicate plaque assays in EK-1 cells as described previously [36]. Using data from 1–4 dpi, viral growth curves were plotted and compared using two-way repeated measures analysis of variance (ANOVA) with interactions, followed by the Benjamini–Hochberg (BH) post-hoc test, all of which were implemented in GraphPad Prism 5.0.

### 2.6. Viral Plaque Area Assay

EK-1 cells were cultured in six-well plates and inoculated with 200 pfu/well of AngHV-1 for 2 h, and then overlaid with culture medium supplemented with carboxymethylcellulose sodium. At different time points post-inoculation, for each strain, 20 randomly selected viral plaques were visualized by indirect immunofluorescent staining with primary antibodies consisting of rabbit polyclonal antiserum raised against AngHV-1 virions. Briefly, cells were fixed in phosphate-buffered saline (PBS) containing 4% (*w*/*v*) para-formaldehyde (PAF) at 4 °C for 15 min and then 20 °C for 30 min. After washing with PBS, samples were permeabilized in PBS containing 0.1% (*v*/*v*) NP-40 at 37 °C for 10 min. Antiserum against AngHV-1 was diluted to 1:1000 in 10% (*v*/*v*) FCS–PBS. Cells were incubated with diluted antiserum for 1 h at 37 °C and washed with 10% (*v*/*v*) FCS–PBS. Secondary antibodies consisted of either Alexa Fluor 488 goat anti-rabbit immunoglobulin G (H + L) or Alexa Fluor 568 goat anti-rabbit immunoglobulin G (H + L) (2 µg/mL; Molecular Probes, Invitrogen) diluted to 1:1000 in 10% (*v*/*v*) FCS–PBS. Cells were incubated with diluted secondary antibody for 30 min at 37 °C. After washing with 10% (*v*/*v*) FCS–PBS and a final wash with PBS, individual plaques were imaged using a Nikon A1R confocal microscope, and areas were measured using ImageJ software [37]. Areas were compared with a repeated measures two-way ANOVA with interactions, followed by the BH post-hoc test. Correlation analyses between viral growth curve data and plaque area data involved the Pearson two-tailed test in GraphPad Prism 5.0.

### 2.7. Retrieval of Cyprinivirus Core Gene Sequences

The protein-coding sequences of four core genes (DNA polymerase, helicase, terminase and uracil-DNA glycosylase) were retrieved from the cyprinivirus genomes listed in Table S1. All cyprinivirus genomes listed in the NCBI Viral Genome Browser (https://www.ncbi.nlm.nih.gov/genome/viruses/) as of 28th February 2020, except molecular clones, were included. In addition, the virus described by Zeng et al. [38] as Crucian carp herpesvirus was also included as a member of the CyHV-2 clade based on sequence similarity. Using a combination of BLASTn (for alignment of two nucleotide sequences) and SnapGene Viewer (v5.1.5), the corresponding core genes were also retrieved from the seven new AngHV-1 genomes sequenced in this study. Among fully sequenced members of the family *Alloherpesviridae* (excluding members of the genus *Cyprinivirus*), ranid herpesvirus 1 (RaHV-1) exhibited the highest average amino acid (AA) BLASTx scores against the CyHV-3 DNA polymerase, helicase and terminase sequences (using sequences from the CyHV-3 strain GZ11 genome in GenBank accession number KJ627438.1). Thus, the corresponding core gene sequences were retrieved from the RaHV-1 full-length genome (GenBank accession number DQ665917.1) for use as an outgroup in the analysis. All retrieved sequences are listed in Table S2.

### 2.8. Analysis of Cyprinivirus Core Gene Sequences

The three core gene sequences retrieved from each cyprinivirus and RaHV-1 were concatenated (order: DNA polymerase, helicase, and terminase). Sets of concatenated sequences were translated to AAs, aligned in MEGA using MUSCLE, and UPGMA phylogenetic trees were generated as outlined above. The resulting bootstrap consensus tree was exported to FigTree, where it was rooted using the outgroup, and trees were transformed into cladograms to aid in the visualization of the topology within species clades. In order to facilitate the application of the best-fitting substitution models and provide more accurate estimations of branch lengths, the same AA alignments were used to generate maximum likelihood (ML) trees (as above). Trees were also generated based on the corresponding codon alignments using the first two codon positions only and subsequently imported into FigTree for further formatting. Using MEGA, each alignment and corresponding ML tree was also used to conduct molecular clock tests based on the same substitution models initially used to generate the tree. ML estimations of transition-transversion bias were generated using MEGA with the same substitution models.

### 2.9. Analysis using Tajima’s Relative Rate Test

For each AA and codon alignment, substitution rates between representative lineages from each species were compared systematically in a pairwise manner using Tajima’s relative rate test [39] based on their distance from the outgroup sequences. Lineages representing each species were selected based on the distance from the root in the species-level ML trees in Figure S1. Random lineages were selected if all branch lengths were equal (or the only available lineage was used in the case of CyHV-1). The tests were implemented in MEGA.

### 2.10. Analysis of Selective Pressure on Codons

Tests for positive selection on codons along branches of interest were conducted using branch-site models in CodeML implemented through pamlX (v1.3.1) [40]. Separate codon alignments were generated based on all cyprinivirus DNA polymerase, helicase, and terminase coding sequences, and these were used to generate three different gene-specific ML trees as outlined above. Branch lengths were converted into substitutions per codon site as described by Brandt et al. [41]. Branches of interest within each tree (exported to Newick format) were labeled using EasyCodeml [42]. Codon alignments were converted into the PAML format using the sequence converter tool in EasyCodeml. Control files for CodeML runs were prepared using pamlX [40], with the following changes to default settings: Model = 2, NSsites = 2, Codon Freq = 2, estFreq = 0, Fix branch length = 2, Fix ω = 0, Fix Alpha = 1, Alpha = 0 and Fix Kappa = 0. For each gene, a second control file for null models was configured identically, except Fix ω = 1 and ω = 1 configurations were used. For each gene of interest, trees (labeled corresponding to branches of interest) and control files were used to generate alternative hypothesis models. Corresponding null hypothesis models were generated in the same way using the second control file (configured with Fix ω = 1 and ω = 1). Alternative and null hypothesis models were compared using log likelihood ratio tests (LRTs) according to the formula 2 × (lnL_1_ − lnL_0_) (or 2ΔlnL), where lnL_1_ is the log of the likelihood value for the alternative hypothesis and lnL_0_ is the log of the likelihood value for the null hypothesis. Using the resulting statistic from the LRT (2ΔlnL), the corresponding *p*-value was derived based on a 50:50 mixture of point mass 0 and $\chi_{1}^{2}$ [43], with degrees of freedom equal to the number of parameters used to estimate the alternative model minus the number used in the estimation of the null model. For each gene, *p*-values were adjusted for multiple comparisons using the BH method via the p.adjust function in R [44].

### 2.11. Estimation of Absolute Divergence Times and Substitution Rates

The datasets of concatenated core gene AA and coding sequences (DNA polymerase, helicase and terminase genes) used in the analyses above were filtered so that they consisted only of unique sequences. These datasets were used to generate new ML trees as outlined above. The new alignments and ML trees were then utilized to generate time trees using the RelTime-ML method [45,46] implemented in MEGA, with time trees generated using the same substitution models used initially to generate each ML tree using the same specified outgroup in all cases. Nodes were calibrated on the basis of various hypotheses regarding possible species divergence times. In the case of co-speciation hypotheses, host divergence times were dated based on estimates generated using the node time search mode on TimeTree.org [47], with confidence intervals used to define lower and upper boundaries of node ages and implemented with uniform calibration densities as described by Mello et al. [48]. Other hypotheses regarding node ages without reasonable lower or upper boundaries were tested using normal calibration densities with 20% standard deviation. All calibrations were entered in units of years. Node age estimates and confidence intervals were retrieved from tabular export files. Substitution rates for specific branches of interest were approximated by dividing the corresponding branch length from the initial ML tree (representing substitutions per site) by the amount of time over which the change was estimated to have occurred (in years), as indicated in each time tree. Thus, the resulting substitution rate estimates were expressed as substitutions per site per year. Estimates were plotted in R using ggpubR [49], thus facilitating comparisons to previous estimates for herpesvirus species [50,51,52].

### 2.12. Comparison of Species-Level Core Gene Diversity among Cypriniviruses and Members of the Family Herpesviridae

Protein-coding sequences of the DNA polymerase, helicase, terminase, and uracil DNA glycosylase genes were retrieved from 10 fully sequenced genomes of 9 mammalian herpesvirus species (Table S1). These represented some of the most frequently sequenced species in the family *Herpesviridae* currently listed in the NCBI Viral Genome Browser. Genomes selected for each species in the family *Herpesviridae* were of diverse geographical origins. The protein coding sequences for the same genes from all CyHV-2, CyHV-3, and AngHV-1 genomes used in analysis above (Table S1) were also included in this comparison. CyHV-1 was not included because only a single genome sequence was available. Taking DNA polymerase, helicase, terminase, and uracil DNA glycosylase protein-coding datasets from each of the 12 viral species separately, these data were used to generate 48 species-level ML trees based on the nucleotide alignments of each gene. These were generated using MEGA as outlined above. Mean nucleotide diversity (π) was estimated on the basis of equation 12.73 in Nei et al. [53] implemented in MEGA with the best-fitting substitution model for each dataset. For each tree, the sum of branch lengths was retrieved from MEGA and divided by the total number of lineages in each tree, giving the mean branch length per lineage. Diversity and mean branch length data were then compared. Since the data were non-normally distributed, as determined using the Shapiro–Wilk test, the impact of key variables on diversity or branch length was assessed using non-parametric two-way testing. This was conducted using either Friedman’s test [54] (if the data facilitated the use of an “un-replicated complete block design”) or alternatively Durbin’s test [55] (if the data required a “balanced incomplete block design”). Post-hoc testing was carried out using a pairwise Wilcoxon test, with *p*-values adjusted for multiple comparison using the BH method. All tests were conducted in R using both the core R stats and PMCR [56] packages. All sequences retrieved for each viral species are listed in Table S2.

## 3. Results and Discussion

### 3.1. Genomic and Biological Comparisons of AngHV-1 Strains

**Figure 1:**
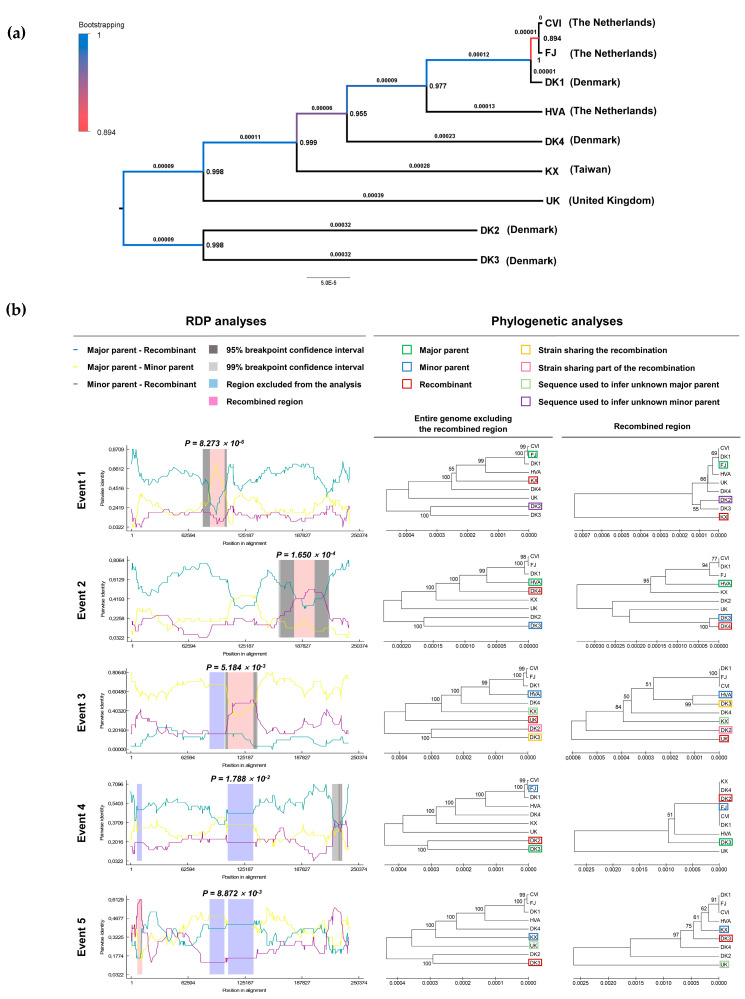
Analysis of AngHV-1 genome sequences. (**a**) Phylogenetic analysis (UPGMA). Bootstrap values (1000 replicates) are indicated at the right of each node. These values are also illustrated by the colors of the branches leading to each node. Numbers above each branch represent substitutions per nucleotide observed along the branch. The geographical origin of each strain is indicated in brackets. (**b**) Recombination analysis. Five potential recombination events were identified using nine sequenced isolates as input. For each event, the left column illustrates the results of RDP analyses, including locations of recombination events and *p*-values. The middle and the right columns show phylogenetic analyses based on the genome excluding the region of recombination and the same tree based on the recombination region only, respectively. Numbers on internal branches indicate bootstrap values (1000 replicates); only values >50% are shown. The scales illustrate the number of substitutions per nucleotide. The color code used is described at the top.

At the start of the present study, there were too few AngHV-1 genome sequences available to facilitate the analysis of genetic diversity among strains. To address this, we generated genomes for seven AngHV-1 strains originating from three geographic regions (Table 1). The phylogenetic comparison of these sequences with the two previously reported genomes revealed sequence identities of >99% and the existence of two major clades (Figure 1a). The two most closely related strains, CVI and FJ, are of similar geographic origin, but in general there was no strict correlation between phylogenetic relationship and geographical origin. Supporting this conclusion, strains isolated in Denmark were distributed throughout the tree, and the strains FJ/CVI and DK1 clustered together despite their different geographical origins.

To explore potential inter-strain recombination, the nine AngHV-1 genomes were analyzed using RDP4 software. Five inter-lineage recombination events were detected that involved the acquisition of genome sections of 16, 22.3, 28, 0.6 and 4.8 kbp (Figure 1b, Table S3). Three of the five recombination events (1, 2 and 5) were supported by more than three detection methods, with others supported by only two methods. Notably, event 1 indicated the involvement of an unknown parent, suggesting the existence of a third, as yet unidentified, lineage. Recombination was also observed between lineages of different geographical origin; for example, event 5 involved recombination between branches leading to strains KX and DK3 (Figure 1b). This observation and the lack of correlation between phylogenetic relationships and geographical origin (Figure 1a) may indicate that the spread of this virus has been connected to anthropogenic factors. However, they may also be explained by an alternative epidemiological scenario in which the mass migration of adult eels to a single geographic region for reproduction facilitates the transmission of AngHV-1 variants originating from geographically distant freshwater regions. Although non-asymptomatic latent carriers are more likely to undertake migration successfully, the added physiological stress of migration and mating may increase the frequency of viral reactivation, which may facilitate co-infection and recombination between strains from different geographical origins. It should be noted that adult eels are believed to die after reproducing [17,18,19]; thus, this hypothesis relies on putative recombinants and their parental strains being transmitted vertically to offspring. However, there is currently no evidence to support vertical transmission, with recent observations suggesting that young (glass) eels seem to reach freshwater free from AngHV-1 [57].

**Figure 2:**
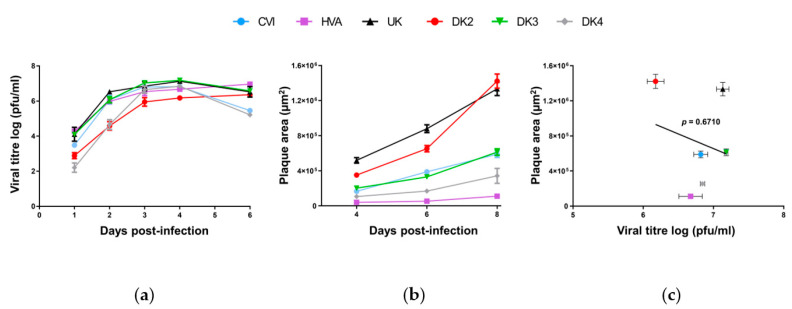
Comparisons of the growth of AngHV-1 strains in vitro. (**a**) Viral growth assay. EK-1 cells were infected with the strains indicated (see top for symbol codes) and the log_10_ value of the titer (pfu/mL) in the cell supernatant was determined at the indicated dpi. Data are presented in Table 1. Cells were infected with the strains indicated, and plaque areas were measured over time. Data presented are the mean ± SEM for measurements of 20 randomly selected plaques. (**c**) Correlation between plaque area measured at 8 dpi (panel (**a**)) and viral titers measured at 4 dpi (panel (**b**)). Data presented are the mean ± SEM.

There are no published comparisons of the biological properties of any sequenced AngHV-1 strains. Despite the high similarity among AngHV-1 genome sequences, strains in more distantly related clades are more likely to exhibit different phenotypic characteristics. We explored this through biological comparisons of representative isolates from each major clade and sub-clade. For each isolate, growth curves and plaque areas were measured in order to compare replication fitness in EK-1 cells. The number of virions produced by strains DK3, DK4, CVI, and UK peaked at 4 days post-infection (dpi), with DK2 and HVA peaking later (Figure 2a). Statistical analyses conducted on data from 1–4 dpi (i.e., before virion production began to decline in some strains) revealed differences between isolates (two-way ANOVA, *p* < 0.0001). Pairwise multiple comparisons revealed consistent significant differences between strains categorized as “fast” (DK3 and UK) and “slow” (DK4 and DK2) replicating strains at each time point, with particularly large differences in titer (mean of up to ~100-fold, *p* < 0.0001) at 2 dpi between these groups. Plaque area assays further supported phenotypic differences between the strains (repeated measures two-way ANOVA, *p* < 0.0001) (Figure 2b). Multiple pairwise comparisons of measurements at 8 dpi revealed that DK2 and UK plaques were significantly larger than those of other isolates (*p* < 0.0001), followed by DK3, CVI, DK4 and then HVA. However, unlike the related cyprinivirus CyHV-3 [36], there was no positive correlation between virion production in the growth curve and plaque area experiments (Figure 2c). Overall, these results demonstrate that the AngHV-1 strains exhibited different abilities to grow in vitro that are unrelated to the clade to which they belong.

All genes in a herpesvirus are assumed to have essential roles in vivo. However, not all genes are required for viral growth in vitro. As a result, such genes may accumulate disabling mutations during growth in a cell culture. The analysis of the seven genomes sequenced in the present study revealed mutations relative to the FJ strain genome, leading to significant disruption in six protein-coding regions (Table 2), thereby indicating that these genes are non-essential in vitro. It is unclear whether some of these mutations are artefacts of viral growth in cell culture. However, the evidence indicates that this is unlikely to be the case with some of these mutations. For example, it is notable that frameshift mutations at the end of ORF102 and ORF112 were detected in most strains. The position of the relevant strains in the phylogenetic tree (Figure 1a) suggested that the haplotype that was assumed to be mutated actually represents the ancestral sequence, and that a monophyletic mutation acquired by a common ancestor of strains CVI-DK1-FJ for ORF102 and strains CVI-DK1-FJ-HVA for ORF112 created these full-length ORFs in strain FJ. For other mutations in Table 2 that are not shared between most strains (or for those that are unique), it is notable that, with the exception of two, all were present in 100% of genomes in their respective populations (raw sequencing data not shown). This suggests that these mutations were not acquired during the two passages of each strain made prior to sequencing. However, the possibility that some of these mutations arose during earlier passaging as part of initial isolation processes cannot be ruled out. In terms of the phenotypes possibly associated with some of these major mutations, it is notable that the two of the most slowly replicating strains (DK2 and DK4) possess the same large deletion at the beginning of ORF15, which encodes a guanosine triphosphatase (GTPase) lacking homologs in other alloherpesviruses [10]. It is not clear whether this deficiency accounts for the slower replication rates of strains DK2 and DK4. These two strains also possess frameshifts in ORF76 (predicted to encode a signal peptide-containing protein) that are different in each strain. Furthermore, both exhibit an additional distinct frameshift elsewhere (Table 2).

### 3.2. Evolutionary Comparison of AngHV-1 and Other Cypriniviruses

**Figure 3:**
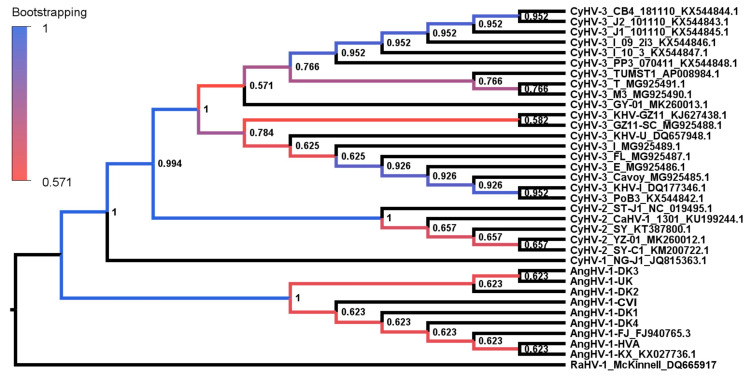
Phylogenetic analysis of concatenated cyprinivirus core gene sequences. Cladogram of bootstrap consensus tree (UPGMA) from phylogenetic analysis of concatenated AA sequences of three core genes (DNA polymerase, helicase and terminase) derived from sequenced cyprinivirus genomes. Bootstrap values (1000 replicates) are indicated at the right of each node. These values are also illustrated through the colors of the branches leading to each node, according to the scale on the top left. Branch lengths are arbitrary.

Given that AngHV-1 is unique among known cypriniviruses in infecting anguillid rather than cyprinid hosts, we reasoned that it might have evolved under significantly different selective forces. To examine the evolution of these viruses, we selected three core genes (DNA polymerase, helicase and terminase) that are among the most conserved genes between all known cypriniviruses and generated phylogenetic trees based on concatenated versions. A bootstrap consensus tree based on the AA sequences indicated that the other cypriniviruses were more closely related to each other than to AngHV-1, with the latter existing as the earliest branch within the genus (Figure 3), which is consistent with previous observations generated using comparable datasets [58]. As indicated by the lower bootstrap values, there is much less confidence associated with the topology within the species clades where there is high sequence similarity among lineages. However, within the CyHV-3 clade, the previously identified European and Asian subclades [36] were observed with high confidence. The topology within the AngHV-1 clade was somewhat similar to that of the genome-based tree in Figure 1a, except for the placement of the UK lineage. Notably, the later cannot be attributed to recombination events described in Figure 1b and Table S3, given the regions involved.

**Figure 4:**
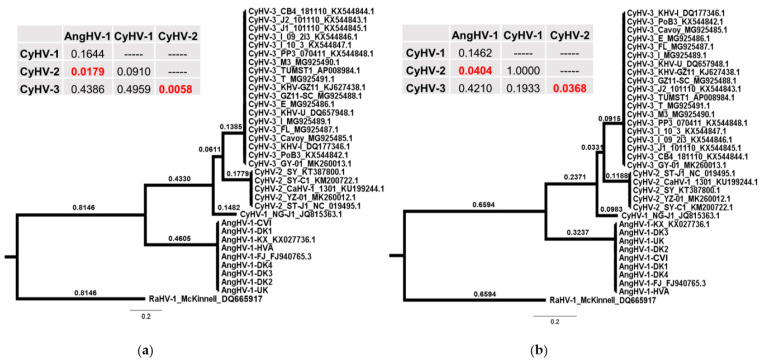
Relative rates of cyprinivirus evolution. ML trees were produced based on the same data used to generate the cladograms in Figure 3. (**a**) ML tree based on concatenated core gene AA sequences. (**b**) ML tree based on concatenated core gene DNA sequences excluding the third codon position. In these trees, branch lengths represent the number of substitutions per site. The results of Tajima’s relative rate tests for pairwise comparison between species clades are presented in each panel. *p*-values highlighted in red indicate significant differences in the rate of evolution between species. The results for Tajima’s relative rate tests presented in panel (**b**) were obtained by considering transversions only. Equivalent tests, considering transitions only, are presented in Table S4.

To provide insight into the fundamental evolutionary differences between cyprinivirus species, additional trees were produced based on the same core gene dataset using AA sequences (Figure 4a) or DNA sequences at the first and second codon positions (hereafter called codon sequences) (Figure 4b). Unlike the bootstrap consensus tree in Figure 3, the branch lengths in the trees in Figure 4 indicate the number of substitutions per site. Both trees indicated that AngHV-1 has accumulated less change in these core genes since divergence from its most recent common ancestor (MRCA) with other cypriniviruses and that CyHV-2 has accumulated more changes. The conclusion that different cypriniviruses exhibit different rates of evolution was supported by molecular clock tests with both AA and codon sequence datasets, which supported the violation of the molecular clock hypothesis, with *p*-values of 1.993 × 10^−12^ and 2.525 × 10^−21^, respectively.

The differences in branch lengths in Figure 4 indicated that there may be varying degrees of support for the violation of the molecular clock hypothesis within the tree. This was investigated further by using Tajima’s relative rate test to conduct pairwise comparisons between lineages occupying different species clades. The analysis of the AA dataset indicated support for the violation of the molecular clock between CyHV-2 and CyHV-3 and also between CyHV-2 and AngHV-1 (Figure 4a), thus also supporting different rates of evolution for these species. Although we observed the expected contrasts in the number of unique substitutions occurring between other species (mostly corresponding to differences in branch length), the lack of support for violations of the molecular clock in these cases was principally due to lower numbers of informative sites. Similar tests were also conducted on the corresponding codon sequence datasets in order to provide further insights into these differences. In most instances, nucleotide substitutions in the first two codon positions will lead to non-synonymous changes, with non-synonymous transversions more likely than transitions to result in non-neutral changes to the biochemical properties of AAs [59,60]. Non-neutral AA substitutions are also more likely to be deleterious and therefore less likely to become fixed in populations unless they are directly advantageous and thus favored through positive selection. Indeed, ML estimates of transition/transversion bias (R) within the dataset indicated R to be 0.70, indicating a bias towards transitions in the first two codon positions. However, corresponding support for molecular clock violations between lineages was exhibited when transversions in the first two codon positions were considered (Figure 4b), but not when transitions were considered (Table S4). As with the AA dataset, these differences were observed between the apparent fastest-evolving lineage, CyHV-2, and two slower-evolving lineages, CyHV-3 and AngHV-1 (Figure 4b). Overall, this indicated that where differences in AA substitution rates between cyprinivirus lineages could be supported, the corresponding codon sequence data suggested that this represented differences in the retention of non-neutral as opposed to neutral AA changes. Given that almost all changes occurring between species are common to all lineages within their respective clades, these differences in substitution rate relate almost entirely to the long-term evolution of these species prior to species divergence.

**Figure 5:**
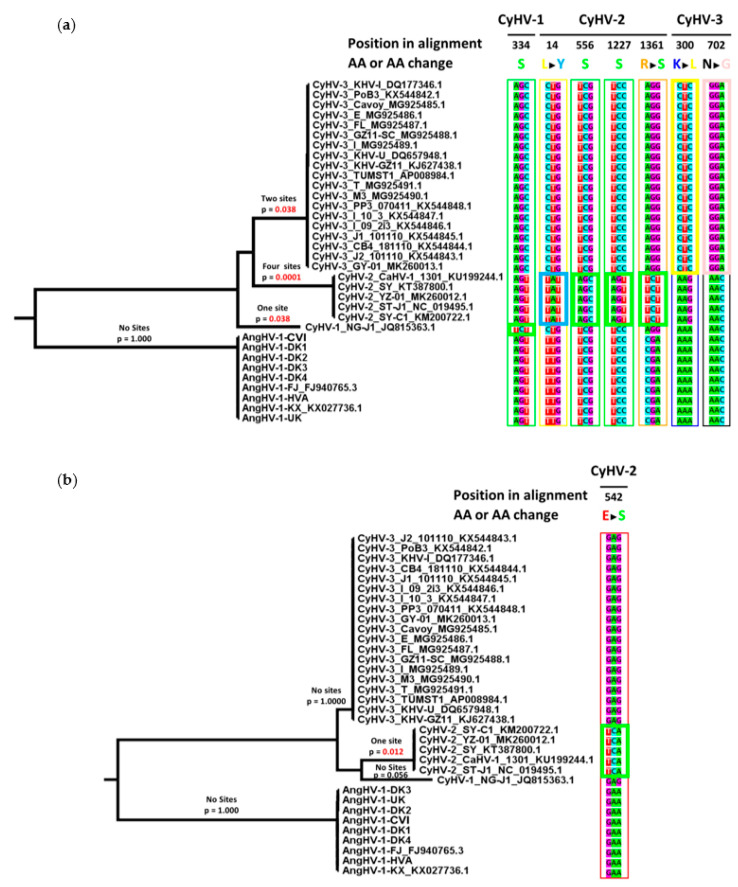
AA changes in cyprinivirus core genes driven by positive selection. CodeML analysis was performed to identify AA changes between cyprinivirus species that were driven by positive selection. Positive selection was identified in the (**a**) DNA polymerase and (**b**) helicase genes. The ML trees based on DNA polymerase and helicase codon alignments are displayed. A summary of results and *p*-values are indicated above each branch of interest. Only changes supported by PP > 0.95 are shown. Codon and AA changes are displayed to the right of each tree, with position in alignments and color-coded descriptions of codon changes indicated on top of each column. Boxes around codons in each column are colored corresponding to the color-coded codon changes on top of each column, with codons representing the changes driven by positive selection indicated by thicker lines. PP values and species-specific positions of AA sites are summarized in Table 3.

The observations from Tajima’s relative rate tests indicated differences in selective pressure acting on cyprinivirus core genes during their evolution. The accumulation of AA substitutions—in particular, non-neutral changes—may have been driven by differences in positive selection [61,62,63]. Adaptive evolution often occurs in a short-lived manner, confined to discrete bursts of change referred to as episodic positive selection, and can be attributed to changes along specific internal branches of trees [61,63,64]. In the context of cyprinivirus phylogeny, and given that each species represents the product of adaptation to a new biological niche, we hypothesized that any episodic selection occurring as part of such adaptation may be observed prior to species divergence in branches leading to each species clade (or lineage in the case of CyHV-1). Focusing on these branches also facilitated the comparison of the selective pressure acting on each species at equivalent periods in their evolutionary history. To establish which of the three core genes may have been under the greatest degree of positive selection within the genus, we tested each gene dataset separately. This was explored using branch-site models [65,66] to test the hypothesis that only some codon changes occurring between cypriniviruses are the products of episodic selection along branches of interest. Taking each gene dataset separately, alternative hypothesis models (permitting positive selection along branches of interest) and null hypotheses models (not permitting positive selection) were compared, thus forming a direct test for the presence of positively selected sites along branches of interest. This strategy resulted in the identification of sites under positive selection in the DNA polymerase and helicase genes (Figure 5). For the DNA polymerase gene, sites under positive selection were identified in the branches leading to the CyHV-2 and CyHV-3 clades and the CyHV-1 lineage (Figure 5a). In the helicase gene, positive selection was detected only along the branch leading to the CyHV-2 clade (Figure 5b). However, with the exception of the branch leading to the CyHV-2 clade in the DNA polymerase dataset, the support for positive selection along other branches was low.

For branches where evidence of episodic positive selection was identified, the corresponding Bayes Empirical Bayes (BEB) output was examined in order to determine the posterior probability (PP)—ranging from 0–1—that given codons evolved under positive selection. The output listed all sites with values of PP >0.5. As expected, for the DNA polymerase gene there were many more sites with values >0.5 for the branch leading to the CyHV-2 clade than in the branches leading to the CyHV-3 clade or the CyHV-1 lineage. Sites with the highest confidence (PP > 0.95) are presented in Table 3 and Figure 5. In agreement with earlier observations from Tajima’s relative rate tests, all AA changes identified as having been driven by positive selection represented instances of non-neutral AA substitutions, with changes to the biochemical properties of the AAs at these positions (Figure 5). Multiple cases of apparent convergent evolution towards serine codons were also observed. The codon sequences in question are very different. With the codon substitution models used, the chances of multiple changes occurring simultaneously within the same codon are extremely small, and it has been shown previously that such changes are driven by positive selection [67]. Thus, it is likely that there were once different AAs encoded at these positions during sequential transition to serine codons. Collectively, as with the observations from Tajima’s relative rate tests, the analysis of positive selection indicated a difference between CyHV-2 and more slowly evolving cypriniviruses, suggesting that faster rates of evolution within these core genes may be linked to (or at least correlated with) differences in selective pressure.

At the other extreme, no evidence for positive selection was identified in branches leading to AngHV-1. Furthermore, using the concatenated dataset, direct comparisons of AngHV-1 to all other species clades in terms of the number of unique substitutions occurring since divergence from the outgroup indicated that AngHV-1 has accumulated fewer changes. This was true for AA substitutions and nucleotide substitutions occurring in the first two codon positions (Table S7). Very similar patterns in AA substitutions were observed when each gene was considered separately (Table S8). Collectively, these observations based on core genes indicate that AngHV-1 may have evolved at a slower rate relative to other cypriniviruses, with lower signals of positive selection. There is currently no evidence indicating that there are additional species that act as natural reservoirs for cypriniviruses. Therefore, in the likely scenario in which these viruses have undergone some degree of long-term co-evolution with their respective current hosts (or previous similar hosts), the differences in their long-term evolutionary rates may have been largely influenced by long-term differences in host behavior or physiology. This may have placed intrinsically different limits on transmission rates and thus how quickly each species has evolved. Indeed, there are several key differences that would favor a higher transmission frequency of other cypriniviruses relative to AngHV-1 and support a lower evolutionary rate for the latter.

Firstly, social behavior such as schooling or shoaling are quintessential cyprinid characteristics [68,69], and carp [70] and *Carassius* ssp. [70,71,72,73] display this behavior (although it is less common with larger adult carp [69]). In contrast, eels are naturally solitary [74], and, although it is possible that they form schools during migration for spawning as an energetically favorable strategy [75], this is considered unlikely [76]. Thus, lower episodes of interaction and greater periods of separation between individuals are likely to reduce the opportunities for AngHV-1 transmission relative to other cypriniviruses. Secondly, both carp and *Carassius* species gather in large numbers to spawn annually throughout their life, possibly even several times in a season [77,78,79,80,81,82,83,84]. In stark contrast, eels will spawn once in their lifetime and then die [19,85,86], making their reproductive behavior less conducive to sustaining continuous viral transmission chains. Thirdly, although clinical outbreaks associated with cypriniviruses are generally restricted by water temperature [12,87], affecting the host physiology and immune response [88], the extent of temperature restriction varies between species. Notably, the temperature requirements for AngHV-1 outbreaks are more restrictive, requiring water temperatures to be >22 °C [14] compared to ~15–28 °C for other cypriniviruses [89,90,91,92,93], thus resulting in less frequent outbreaks of AngHV-1 disease. Overall, the possibility of greater transmission opportunities among other cypriniviruses compared to AngHV-1 is consistent with our observations, indicating that AngHV-1 may have accumulated fewer changes since the divergence from the MRCA of cypriniviruses. Although the long-term evolution of cypriniviruses may have been influenced by the natural factors described above, adaption to and propagation within aquaculture environments may play a more prominent role in the recent evolutionary history of these viruses.

### 3.3. Absolute Estimates of Substitution Rates and Divergence Times for Cypriniviruses

**Figure 6:**
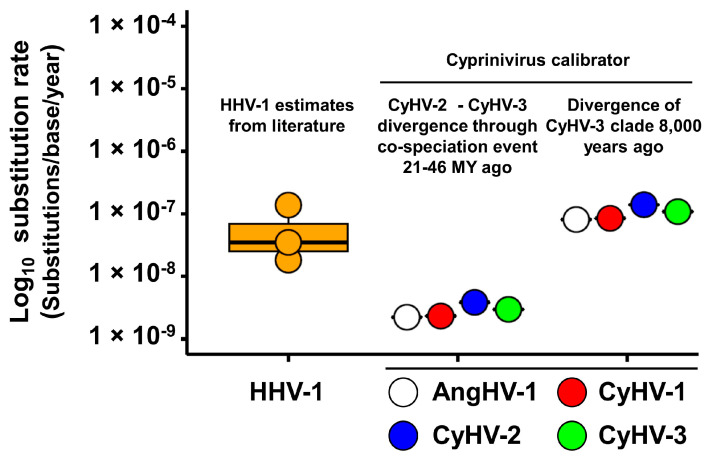
Comparison of species-specific nucleotide substitution rate estimates between HHV-1 and cypriniviruses. HHV-1 substitution rates were reported in the studies described in Table S9. Rate estimates for cypriniviruses were estimated based on two different calibration hypotheses. Corresponding time trees are available in Figure S3.

The results presented above (Figure 4) support the existence of differences in evolutionary rates between cypriniviruses. To expand on this, we explored the possibility of generating absolute estimates of evolutionary rates (in substitutions/site/year) and divergence times (in years) for each virus. Such estimates are often generated by assigning ages to internal nodes of phylogenetic trees (known as node calibration), using this to extrapolate node ages and branch substitution rates elsewhere in the tree [45,48]. When applying this approach to phylogenetic trees of related viruses that infect related hosts, node calibration often relies on the assumption of host–virus co-speciation [94]. Although many members of the family *Herpesviridae* seem to have evolved through co-speciation with their hosts [95], it has been previously proposed that co-speciation has not been as prevalent in the family *Alloherpesviridae*, most notably in the context of the genus *Cyprinivirus* [58]. For example, the phylogenetic relationship between AngHV-1 and other cypriniviruses is much closer than the relationship between their hosts, suggesting that one of these lineages emerged via a host jump. Based on the phylogenetic trees for the cypriniviruses (Figure 3 and Figure 4), the only remotely plausible co-speciation event would be between CyHV-2 and CyHV-3, corresponding to the divergence of the genera *Cyprinus* and *Carassius*, which is estimated to have occurred 21–46 million years ago (MYA) (Figure S2). Interestingly, this scenario is also compatible with CyHV-2 and CyHV-3 emerging in their current hosts through a host jump from other members of the same host genus, similar to a hypothesis proposed previously elsewhere [96]. This hypothesis was explored further by generating time trees calibrated under the assumption of co-speciation (Figure S3) in order to establish whether such a scenario would result in plausible evolutionary rates and species divergence times. Under this scenario, the mean approximated AA substitution rate for cypriniviruses was estimated to be 4.37 × 10^−9^ substitutions/site/year (Table 4). This is comparable to the often-cited mean AA substitution rates for members of the family *Herpesviridae* of 3.0 × 10^−9^ [95] and 4.4 × 10^−9^ [97] substitutions/site/year. In order to generate approximations of the corresponding nucleotide substitution rates, the same time trees were generated based on codon alignments (excluding the third base). This resulted in a mean rate estimate for cypriniviruses of 2.84 × 10^−9^ substitutions/base/year, in close agreement with mean rates for various members of the family *Herpesviridae* (also excluding the third base in codons), estimated to be 2.7 × 10^−9^ substitutions/site/year [97]. We compared these to species-specific and more recently obtained substitution rate estimates for human alphaherpesvirus 1 (HHV-1) that were also generated through internal node calibration (Table S9). Although estimates for HHV-1 vary considerably, the corresponding estimates for cypriniviruses based on this co-speciation scenario were much lower (Figure 6).

Under this scenario, lineages within AngHV-1 and CyHV-2 species clades are estimated to have diverged at similar times ~20,000–30,000 years ago, respectively, based on AA data. Using the same data, lineages within the CyHV-3 clade are estimated to have diverged ~180,000 years ago (Table 4 and Figure S3). Somewhat comparable estimates were observed using the codon dataset (Table 4 and Figure S3). However, it is necessary to consider the possibility that the emergence and evolution of some or all of these cypriniviruses was, at least in part, driven by aquaculture activities. Indeed, this has been speculated to be the case with CyHV-3 [96,98] and many other pathogens of farmed fish species [98,99,100,101,102]. Even in a scenario in which such anthropogenic factors did not lead to the divergence of separate viral species but instead to the selection of increasingly pathogenic strains within aquaculture environments, within each species, it would be expected that the MRCAs of contemporary pathogenic isolates would not have existed prior to the commencement of the earliest human aquaculture. On this topic, the earliest estimates of human aquaculture date back to the Neolithic era, over 8000 years ago [103], incidentally involving the CyHV-3 and CyHV-1 host species *Cyprinus carpio*. These estimates may indicate that either co-speciation could not have occurred or that estimations of host divergence times are inaccurate. Alternatively, very early divergence times (prior to aquaculture) may indicate that, within each species, there were multiple separate adaptations to aquaculture environments much later.

The node ages generated from calibrations made on the basis of the co-speciation assumption are very ancient, and it is important to consider the impact that this alone may have on subsequent estimates. For example, the timescales over which estimates are based will have a substantial impact on node age and rate estimates due to the time-dependent rate phenomenon (TDRP) [94,104,105,106,107]. In particular, the use of very ancient calibration dates on deep nodes may inherently overestimate the age of shallower nodes [104,108]. Thus, even if the co-speciation hypothesis is correct, given the calibration constraints it imposes, it may be intrinsically difficult to reconcile estimates derived from this process with comparatively recent events such as adaptation to aquaculture.

Doubts surrounding the plausibility of these estimates are mainly predicated on the idea that contemporary species divergence events correspond directly to adaptation to aquaculture environments. However, it is unclear whether this scenario could itself produce plausible cyprinivirus substitution rate estimates. The earliest known evidence of *Cyprinus carpio* aquaculture (dating back 8000 years) may represent the earliest possible date of cyprinivirus adaptation to aquaculture environments. Additional time trees were generated on the basis of the MRCA of CyHV-3 lineages being of this age. In this scenario, lineages within the AngHV-1 and CyHV-2 species clades are estimated to have diverged ~500–1000 years ago, respectively, based on AA data (with comparable estimates from codon data) (Table 4). However, these divergence times result in much higher AA and nucleotide substitution rates (Table 4), with the latter (mean of 1.04 × 10^−7^ substitutions/base/year) being within the range of HHV-1 estimates (Figure 6). However, given that the environmental constraints on cyprinivirus lytic infection (or possible reactivation from latency) result in corresponding constraints on transmission rates, we propose that these estimates may be implausibly high. Furthermore, even if potential increases in transmission rates for some cypriniviruses have been facilitated by the increased scale and intensity of aquaculture activities, this would represent a very recent phenomenon that may not yet have had such a high impact on the rates at which the resulting mutations became fixed in populations. This may mean that the adaptation of CyHV-3 to aquaculture environments occurred significantly earlier than 8000 years ago or that divergence was earlier and independent of aquaculture activities. Although the evolutionary rate estimates resulting from the co-speciation scenario are more plausible, given (i) the possible influence of very ancient calibration dates, (ii) assumptions surrounding co-speciation itself, and (iii) uncertainty surrounding the dating of host divergence times, there remains a high degree of imprecision. Therefore, further exploration by the application of alternative higher-confidence calibration strategies will ultimately be necessary.

In this regard, the generation of short-term estimates using time-structured data may be a useful alternative avenue and has been successfully applied elsewhere [109,110]. This approach does not rely on speculative assumptions regarding calibration, as temporal information is derived directly from sequence sampling dates. This approach typically results in much earlier divergence times and higher evolutionary rates compared to node calibration approaches due to TDRP. This may be due to a lag in purifying selection leading to the incomplete removal of deleterious or transient variants from datasets. Thus, short-term estimates may be more representative of non-lethal mutation rates than rates at which these mutations become fixed in populations (i.e., substitution rate) [105,107,111]. However, these estimates may be useful for understanding patterns of diversity and comparing the impact of recent epidemiological changes (such as adaptation to aquaculture facilities) between viral species. Indeed, if currently studied cyprinivirus strains and associated phenotypes have mostly emerged through selection within aquaculture settings, short-term estimates may be of more scientific relevance. Thus, collaborative efforts towards generating the necessary sample sizes required for such analysis may be beneficial for the field in general.

### 3.4. Comparison of Core Gene Diversity Between Cypriniviruses and Members of the Family Herpesviridae

**Figure 7:**
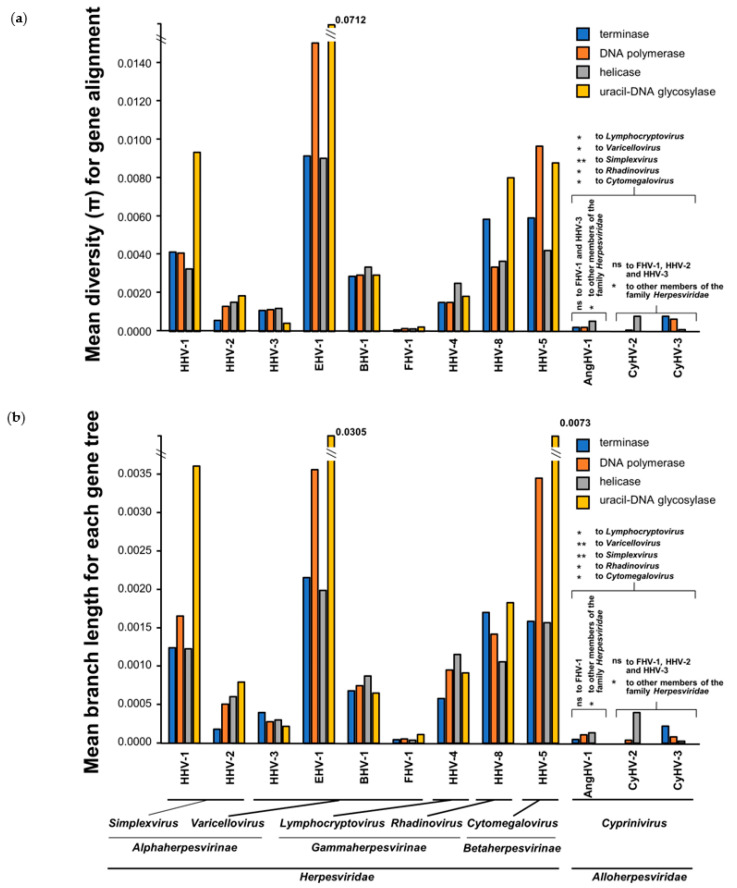
Comparison of nucleotide diversity in core genes between cypriniviruses and members of the family *Herpesviridae*. Comparisons were based on species-level nucleotide alignments and trees generated using DNA polymerase, helicase, and terminase sequences from each species. Sequences from a fourth core gene, uracil-DNA glycosylase, were also added, facilitating the comparison of diversity between highly conserved and less well conserved core genes. All species name acronyms are defined in Table S1. The comparison consisted of 492 sequences from 123 sequenced strains. The 48 phylogenetic trees corresponding to each species-level gene alignment are provided in Figures S4 and S5. (**a**) Diversity (π) from each species-level nucleotide sequence alignment. (**b**) Mean branch length for each species-level tree. Pairwise comparison: * = *p* < 0.05, ** = *p* < 0.01.

We also used the additional AngHV-1 genomes generated in this study to gain insight into the diversity existing within the AngHV-1 species clade relative to that of closely related cypriniviruses and distantly related species within the family *Herpesviridae*. The three core cyprinivirus genes used throughout this study (DNA polymerase, helicase, and terminase) share functions with core genes of well-characterized species of the family *Herpesviridae*, and thus these sets of genes presented an ideal basis for such comparisons. Sequences from a fourth core gene, uracil DNA glycosylase, were also included, facilitating the comparison of diversity between both highly conserved and less well conserved cyprinivirus core genes. Accordingly, we retrieved protein coding sequences from the same four core genes from some of the most frequently sequenced species of the family *Herpesviridae* (with 10 randomly sequenced genomes from each species) and compared their species-level sequence diversity to that of their functional homologs from cyprinivirus species (Figure 7). Although this sample of genomes taken from each species of the family *Herpesviridae* is unlikely to capture the full extent of diversity within each species clade, the relative differences in diversity were in agreement with previous reports [112,113,114,115], with felid alphaherpesvirus 1 (FHV-1), human alphaherpesvirus 2 (HHV-2), and human alphaherpesvirus 3 (HHV-3) exhibiting relatively low diversity (Figure 7). In general, core gene nucleotide diversity (π) and mean branch length estimates were much lower among cyprinivirus species than among species of the family *Herpesviridae* (Figure 7). The DNA polymerase, helicase, and terminase play crucial roles in DNA metabolism and genome replication in both groups of viruses [6,9] but appear to be more consistently exposed to strong purifying selection within the cyprinivirus species. These are also among the most conserved core genes between these species [9,10]. Uracil-DNA glycosylase also plays key roles in herpesvirus replication [116,117], with a role in maintaining genome sequence fidelity [118], but it is considered auxiliary in vitro [119]. Furthermore, in addition to not being as highly conserved between cypriniviruses as the other three core genes, uracil DNA glycosylase appears to be absent from other members of the family *Alloherpesviridae*. Despite this, uracil DNA glycosylase appears to be under even greater purifying selection within each cyprinivirus species relative to the other three core genes. With the exception of a single AA insertion/deletion near the end of the uracil-DNA glycosylase of the European and Asian lineages of CyHV-3, there are no other changes in uracil-DNA glycosylase sequences among lineages within cyprinivirus species clades. This strong purifying selection possibly indicates important species-specific roles of this protein in cypriniviruses. Similar observations were not made for this gene among species of the family *Herpesviridae* (Figure 7).

To examine these differences further, statistical comparisons of diversity were made between all species, using data from the four core genes (Figure 7). Controlling for the intrinsic variability between species, none of the genes exhibited a significant variation in diversity relative to each other (*p* > 0.05). There were significant differences in diversity between genera even when controlling for the apparent large differences between the families *Alloherpesviridae* and *Herpesviridae* in Figure 7a (*p* < 5 × 10^−4^). Further examination by pairwise comparison revealed significant differences in diversity between the cypriniviruses and all other genera of the family *Herpesviridae* tested (*p* < 0.05, Table S10). However, there was less support for differences in diversity between individual cypriniviruses and low-diversity species of the family *Herpesviridae* such as FHV-1, HHV-2, and HHV-3 (Figure 7a, Table S11). Similar results were observed for mean branch length comparisons (Figure 7b, Tables S10 and S11). Furthermore, comparisons among cypriniviruses revealed no differences between them, with all exhibiting similarly low diversity.

These observations may provide interesting insights into the differences between the two distantly related viral groups. The low diversity observed among contemporary cypriniviruses relative to diversity among species in the family *Herpesviridae* may seem counterintuitive given the greater virulence exhibited by cypriniviruses [87], their global distribution, and in particular considering the ease of propagation within aquaculture facilities due to high stocking densities and subsequent close contact between hosts. However, these differences may not only reflect the fundamental differences in biology but also the epidemiological circumstances linked to anthropogenic influence and possibly a certain amount of methodological bias. Firstly, it is likely that recent anthropogenic activities, including international trade associated with increased globalization, have played a significant role in the rapid spread of cypriniviruses [120,121,122,123,124,125,126], and thus their widespread distribution may only be a very recent phenomenon. Secondly, the sequence data in the present study may be biased towards pathogenic strains that have successfully adapted to aquaculture environments and are responsible for severe clinical outbreaks, thus leading to underestimations of cyprinivirus diversity. For example, the epidemiology and diversity of CyHV-3 may be very different in the wild, where environments are more heterogeneous and high levels of virulence are not as easily maintained [127,128,129]. Thirdly, if such sampling bias exists, the resulting low diversity may be compounded by the fact that modern aquaculture settings represent a very recently encountered biological niche, with very little time for viral strains to accumulate diversity. In addition, the high degree of standardization in modern aquaculture practices may act to limit diversity and essentially contribute towards a genetic bottleneck effect. Fourthly, relative to the family *Herpesviridae*, the replication of cypriniviruses is highly dependent on environmental factors; specifically, temperature [14,90,91,92]. Given that short-term evolutionary estimates provide a good indication of the rate at which non-lethal mutations emerge, differences between species in this regard may be related directly to differences in diversity, while also taking into account differences in sampling windows. Thus, comparisons of estimates between cypriniviruses and species of the family *Herpesviridae* may provide much more useful insights into differences in diversity between these distantly related groups.

## 4. Conclusions

The present study provides insights into previously unknown characteristics of the AngHV-1 species phylogeny, including the degree of phenotypic diversity and the potential role of recombination in evolutionary outcomes. In summary, (i) the AngHV-1 strains are characterized by a low genetic diversity; (ii) despite low diversity, there is still evidence of recombination between strains; (iii) phylogenetic relationships and recombination events do not correlate with geographic origin; (iv) biological properties differ among strains, although it is unclear whether the associated mutations occurred naturally or during isolation; and (v) the presence of disrupted genes indicates that these genes are non-essential for replication under the in vitro experimental conditions used. In this work, we have demonstrated the importance of coupling genomic and biological comparisons of viral strains, providing a firm basis from which to explore the connections between genotype and phenotype in vitro and in vivo.

Our investigations into evolutionary rates and selective pressures acting on selected core genes facilitated a greater understanding of AngHV-1 evolution in the context of the genus *Cyprinivirus*. We observed that AngHV-1 core genes have accumulated less change relative to other cypriniviruses, and that the greater accumulation of substitutions among other species may be driven by differences in positive selection. These findings are also consistent with the expected differences in epidemiological circumstances between AngHV-1 and other cypriniviruses, which infect very different host species that have very different lifecycles and behaviors.

After a thorough exploration, we concluded that the estimation of cyprinivirus divergence times and evolutionary rates based on node calibration approaches is currently problematic. This may be due principally to the limited calibration options and also due to the speculative assumptions and high degree of uncertainty surrounding node calibration hypotheses. As a result, we recommend collaborative efforts towards generating estimates of evolutionary rates over recent, shorter timescales. This would represent an important step towards understanding how recent epidemiological changes, such as propagation within aquaculture environments, have influenced the evolution of AngHV-1 and other cypriniviruses.

We further exploited the AngHV-1 genome data by comparing the diversity of core gene sequences among lineages within each cyprinivirus species clade. This revealed no difference in diversity between cypriniviruses in this respect. However, cyprinivirus core gene diversity was generally much lower than that observed for individual members of the family *Herpesviridae*. Although this may reflect fundamental underlying differences in biology that dictate transmission rates or rates of change between these distantly related viruses, we conclude that anthropogenic factors, sampling bias, and the much smaller sampling windows for cypriniviruses may also contribute towards these observations. We conclude that the comparison of short-term evolutionary rates between cypriniviruses and members of the family *Herpesviridae* may provide useful insights into differences in core gene diversity between these two distantly related viral groups.

## Figures and Tables

**Table 1:** List of AngHV-1 strains referred to and used in this study.

Short Name Used in This Study	Original Name	Geographic Origin	Host	GenBank Accession Number	References
CVI ^1^	CVI NL 500138	The Netherlands	*Anguilla anguilla*	MW580849	[10]
HVA ^1^	HVA486123			MW580854	[14,24]
UK ^1^	UK80	United Kingdom		MW580855	-
DK1 ^1^	DK-200249	Denmark		MW580851	-
DK2 ^1^	DK-2008-50-66-1			MW580850	-
DK3 ^1^	DK-205223-2			MW580852	-
DK4 ^1^	DK-206116-1			MW580853	-
FJ		The Netherlands		FJ940765.3	[10]
KX		Taiwan	*Anguilla japonica*	KX027736.1	[21]

^1^ The full-length genome sequences of these strains were generated in the present study.

**Table 2:** Gene disruptions identified in AngHV-1 isolates relative to reference strain FJ.

Strain	ORF ^1^					
**CVI**		25 ^3^				
**DK1**						
**HVA**					102	
**DK4**	(15) ^2^	(25) ^3^		(76) ^3^	102	112 ^4^
**KX**					102	112 ^5^
**UK**					102	112 ^5^
**DK2**	(15) ^2^		(53)	(76) ^3^	102	112 ^4^
**DK3**					102	112 ^4^

^1^ Numbers that are in brackets represent ORFs with at least one frameshift near the beginning or in the central part of the ORF. Numbers that are not in brackets represent ORFs with a frameshift near the end of the ORF or other partial disruptions. Numbers that are underlined indicate the existence of a sub-population (representing the minority) that does not exhibit a disruption relative to the FJ isolate. ^2^ Five base pair insertion in 5’ UTR followed by 244 bp deletion including the first 234 bp of ORF15 in FJ strain ^3^ Disruptions to these ORFs are different in each strain. ^4^ One base pair deletion creating a frameshift, with reading frame subsequently restored by 1 bp insertion 83 bp downstream. ^5^ These strains have the same initial 1 bp deletion in ORF112 as described in footnote 4 above but without the compensating 1 bp insertion 83 bp downstream leading to a premature stop codon relative to other isolates.

**Table 3:** Summary of AA changes identified as having been driven by positive selection.

Gene	Viral Species	Position in Alignment ^1^	AA Sites Under Positive Selection (Location in Relevant Species)	Bayes Posterior Probability ^3^
DNA polymerase	CyHV-1	334	314S ^2^	0.963
	CyHV-2	14	14Y	0.964
		556	510S ^2^	0.962
		1227	1150S ^2^	0.966
		1361	1282S	0.965
	CyHV-3	300	282L	0.976
		702	656G	0.951
helicase	CyHV-2	542	508S	0.959

^1^ AA alignments for cyprinivirus DNA polymerase and helicase genes are available in Tables S5 and S6. ^2^ Likely to represent convergent evolution towards serine codon. ^3^ Only sites with PP > 0.95 are reported. These are also displayed in Figure 5.

**Table 4:** Summary of species clade divergence time estimates and species-specific substitution rates generated as part of two different calibration hypotheses.

Calibration Hypotheses ^1^	Species	AA		Codon (Position 1 + 2)	
		Divergence Time (In Years) and Confidence Intervals	Substitution Rate (Per Site Per Year) ^2^	Divergence Time (In Years) and Confidence Intervals	Substitution Rate (Per Base Per Year) ^2^
Divergence of CyHV-2 and CyHV-3 21–46 MYA	CyHV-1	N/A	3.48 × 10^−9^	N/A	2.33 × 10^−9^
	CyHV-2	26,735 (16,980–42,092)	5.96 × 10^−9^	9348 (7420–11,777)	3.85 × 10^−9^
	CyHV-3	181,183 (91,633–358,249)	4.81 × 10^−9^	292,158 (233,744–365,171)	2.97 × 10^−9^
	AngHV-1	23,341 (11,091–49,123)	3.22 × 10^−9^	16,657 (8138–34,096)	2.23 × 10^−9^
	Mean	N/A	4.37 × 10^−9^	N/A	2.84 × 10^−9^
Divergence of CyHV-3 clade 8000 years ago	CyHV-1	N/A	7.87 × 10^−8^	N/A	8.47 × 10^−8^
	CyHV-2	521 (420–647)	1.37 × 10^−7^	256 (139–470)	1.40 × 10^−7^
	CyHV-3	8000 (6400–10,000)	1.09 × 10^−7^	8000 (6570-9741)	1.08 × 10^−7^
	AngHV-1	1032 (918–1160)	7.29 × 10^−8^	456 (223–934)	8.11 × 10^−8^
	Mean	N/A	9.93 × 10^−8^	N/A	1.04 × 10^−7^

^1^ Corresponding time trees are presented in Figure S3. ^2^ These represent changes along branches leading to each species clade (or single lineage in the case of CyHV-1) and thus represent species-specific substitution rates; i.e., the rates at which mutations became fixed in populations prior to species clade divergence.

## Data Availability

Not applicable.

## Supplementary Materials

The following are available online at https://www.mdpi.com/article/10.3390/microorganisms9050998/s1, Figure S1: ML phylogenetic trees for cypriniviruses based on AA alignments of three core genes (DNA polymerase, helicase and terminase), Figure S2: Output from the Node Time tool (timetree.org) for estimating the divergence time between the genera *Cyprinus* and *Carassius*, Figure S3: Cyprinivirus time trees, Figure S4: Species-level phylogenetic trees based on nucleotide alignments of core gene sequences from cypriniviruses, Figure S5: Species-level phylogenetic trees based on nucleotide alignments of core gene sequences from members of the family *Herpesviridae*, Table S1: Strains used in the present study, Table S2: Sequences retrieved from GenBank and generated locally that were used in the present study, Table S3: Summary of recombination events among AngHV-1 strains, Table S4: *p*-values from Tajima’s relative rate test based on transitions in the first two codon positions, Table S5: Alignment of cyprinivirus DNA polymerase codon sequences, Table S6: Alignment of cyprinivirus helicase codon sequences, Table S7: Supplementary output from Tajima’s relative rate tests, Table S8: AA changes in individual core genes between AngHV-1 and other cypriniviruses, Table S9: Details of HHV-1 nucleotide substitution rate estimates reported elsewhere, Table S10: Pairwise comparisons (Wilcoxon test) of gene diversity between each genus, Table S11: Pairwise comparisons (Wilcoxon test) of gene diversity between each species.
