# Supplementary figures and images for: Genomes of Anguillid Herpesvirus 1 Strains Reveal Evolutionary Disparities and Low Genetic Diversity in the Genus Cyprinivirus
Source: Microorganisms. 2021 May 5;9(5):998. doi: 10.3390/microorganisms9050998 (PMC8148134; doi:10.3390/microorganisms9050998)

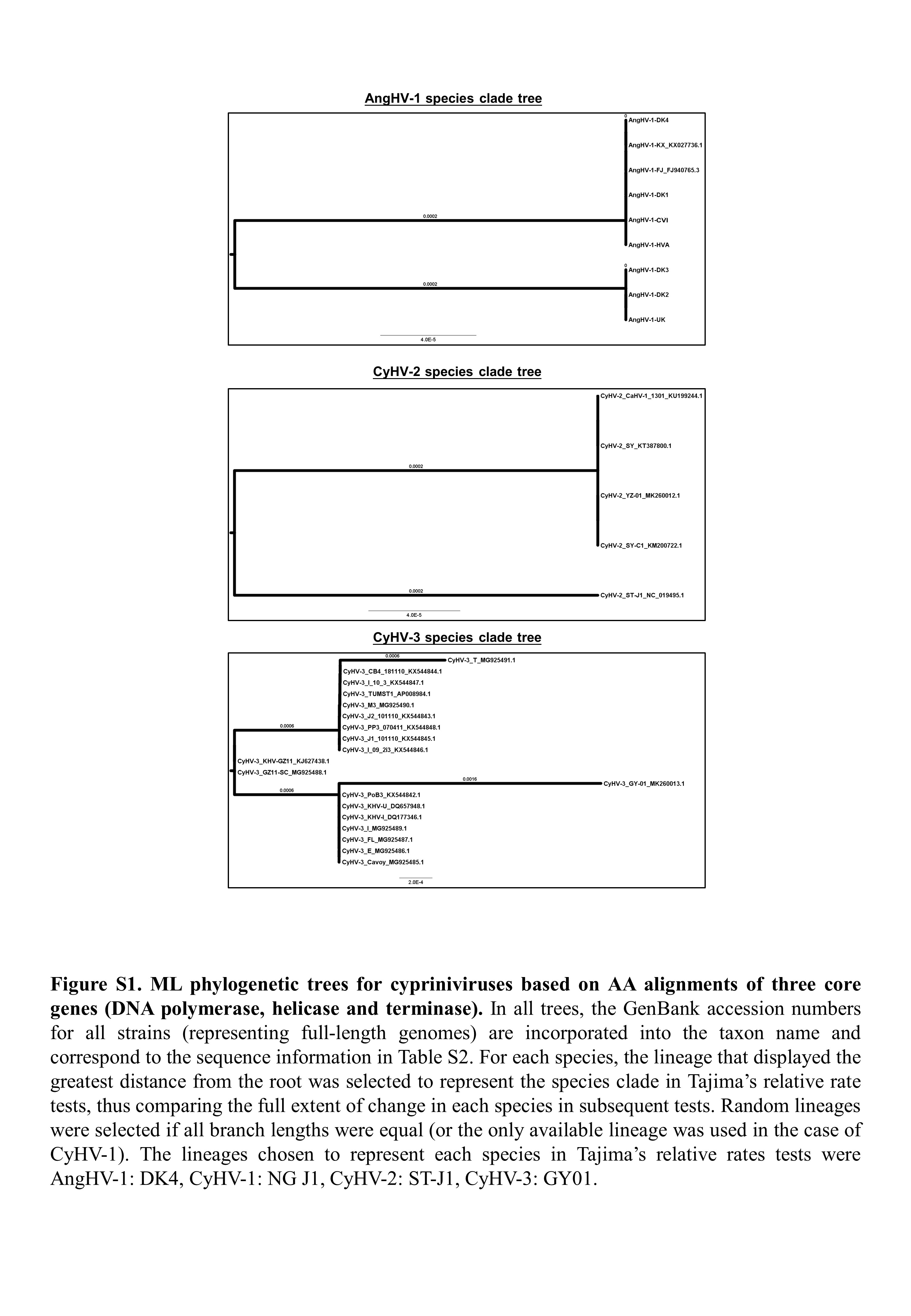

Supplement: Supplementary file 1 [file microorganisms-09-00998-s001.zip › Supplementary Figures S1 FINAL.jpg]

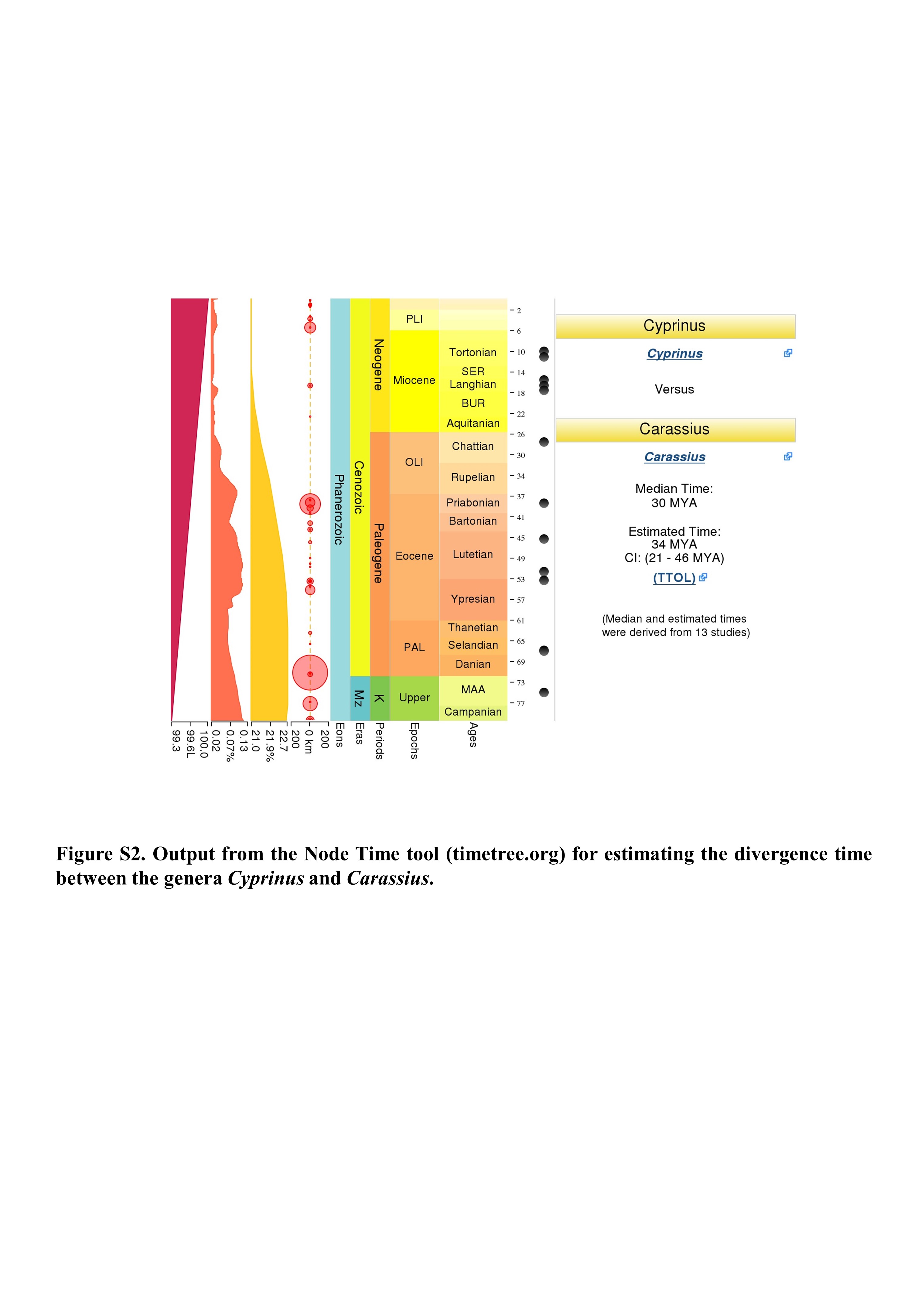

Supplement: Supplementary file 1 [file microorganisms-09-00998-s001.zip › Supplementary Figures S2 FINAL.jpg]

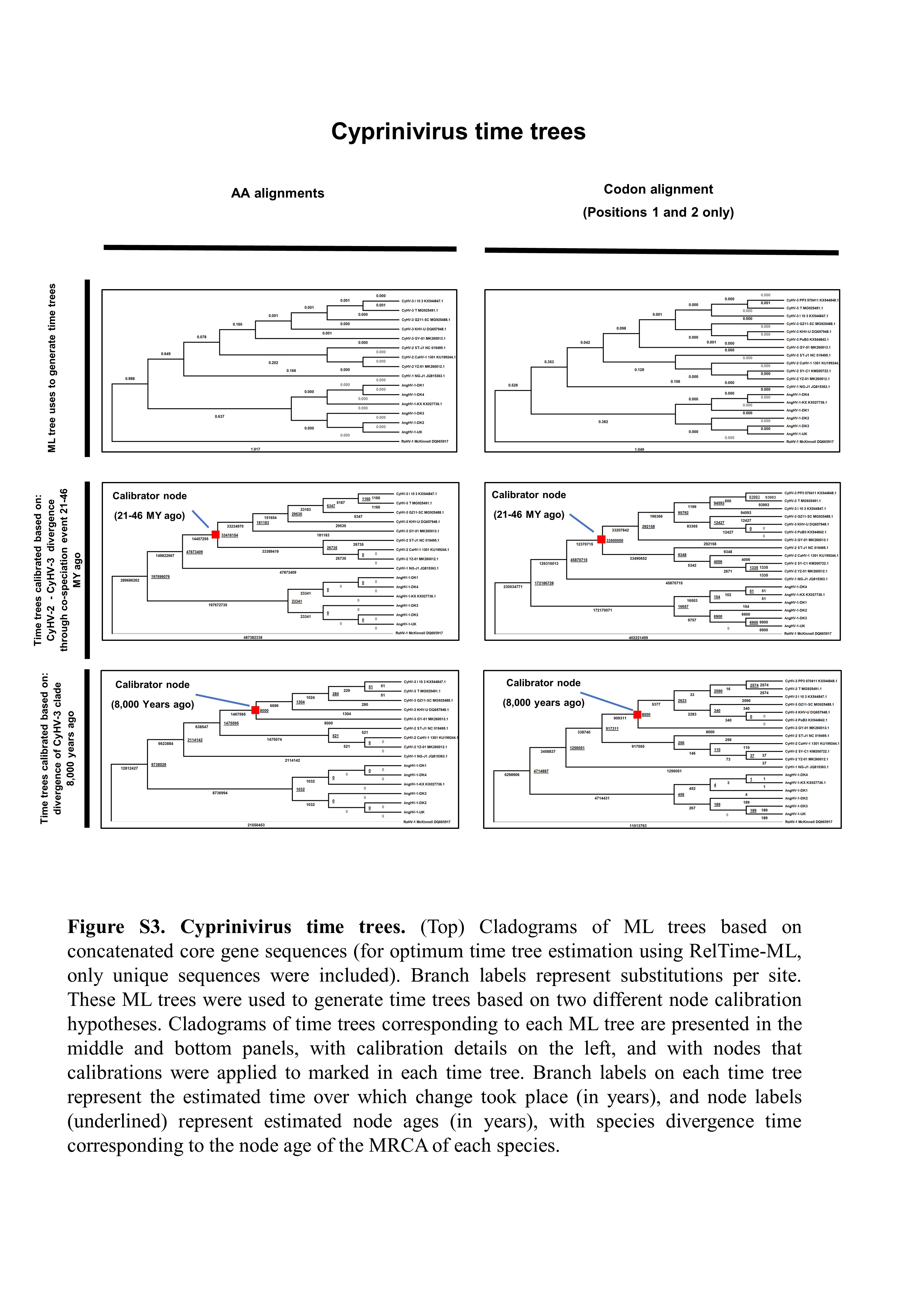

Supplement: Supplementary file 1 [file microorganisms-09-00998-s001.zip › Supplementary Figures S3 FINAL.jpg]

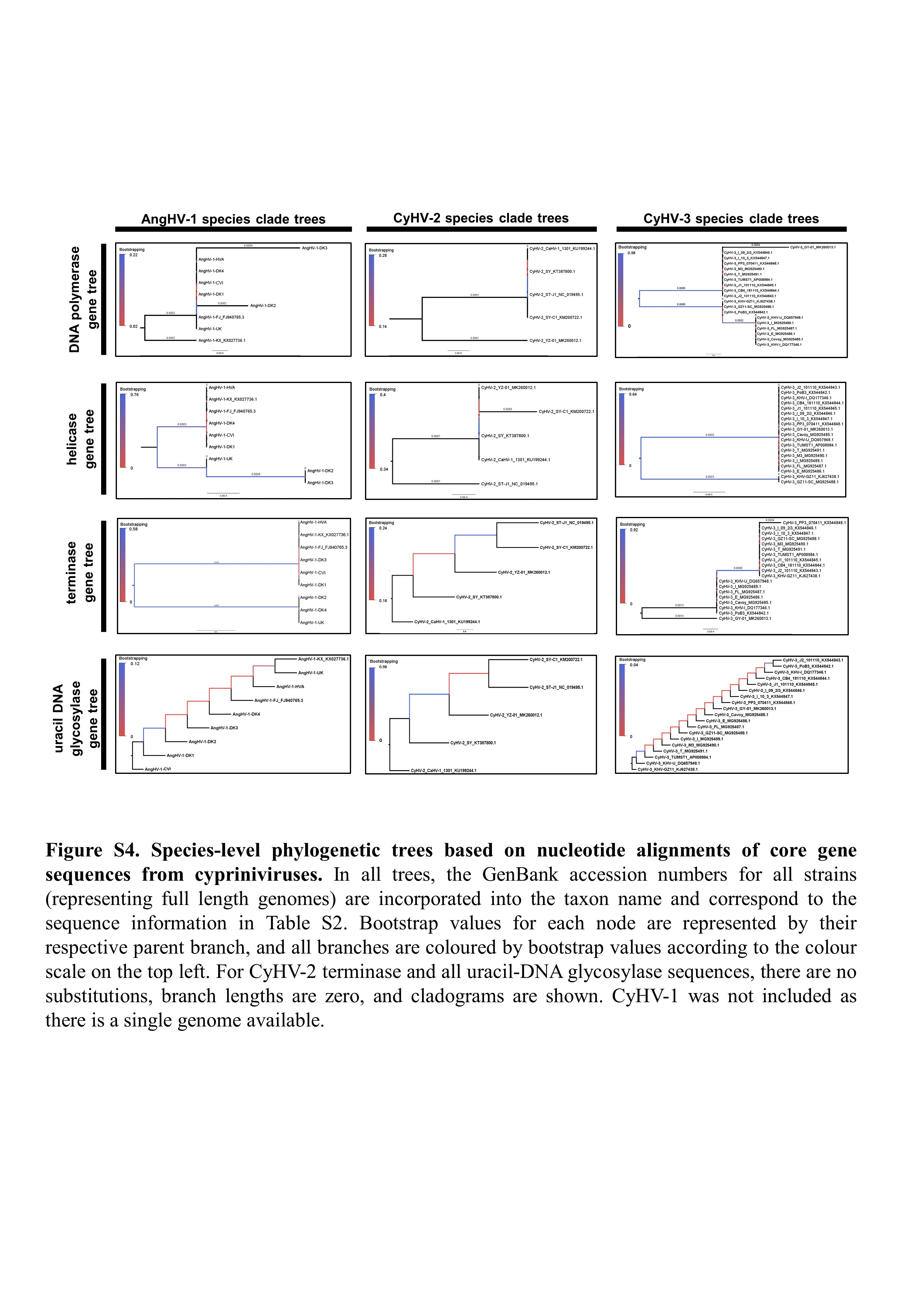

Supplement: Supplementary file 1 [file microorganisms-09-00998-s001.zip › Supplementary Figures S4 FINAL.jpg]

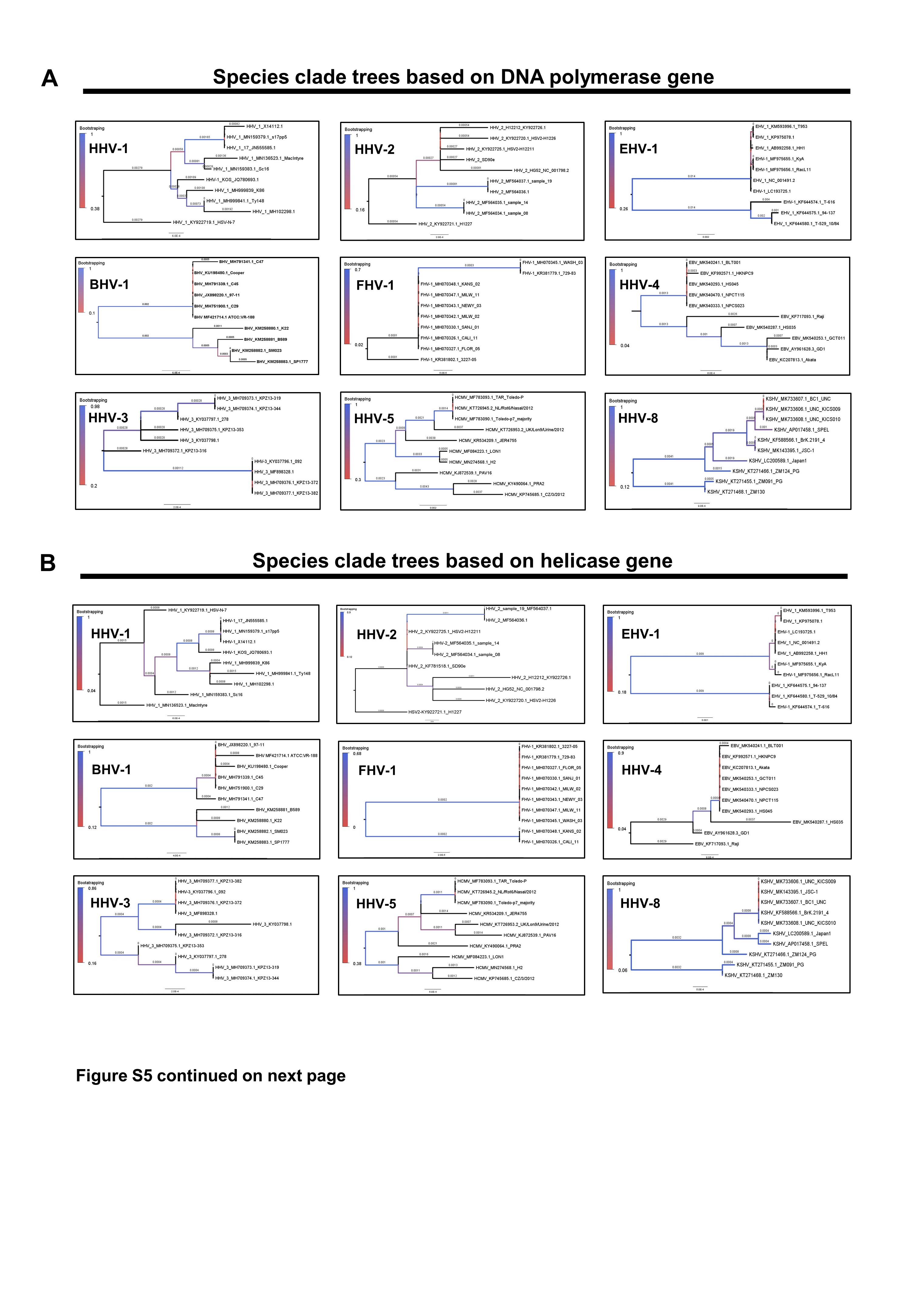

Supplement: Supplementary file 1 [file microorganisms-09-00998-s001.zip › Supplementary Figures S5 FINAL Part 1.JPG]

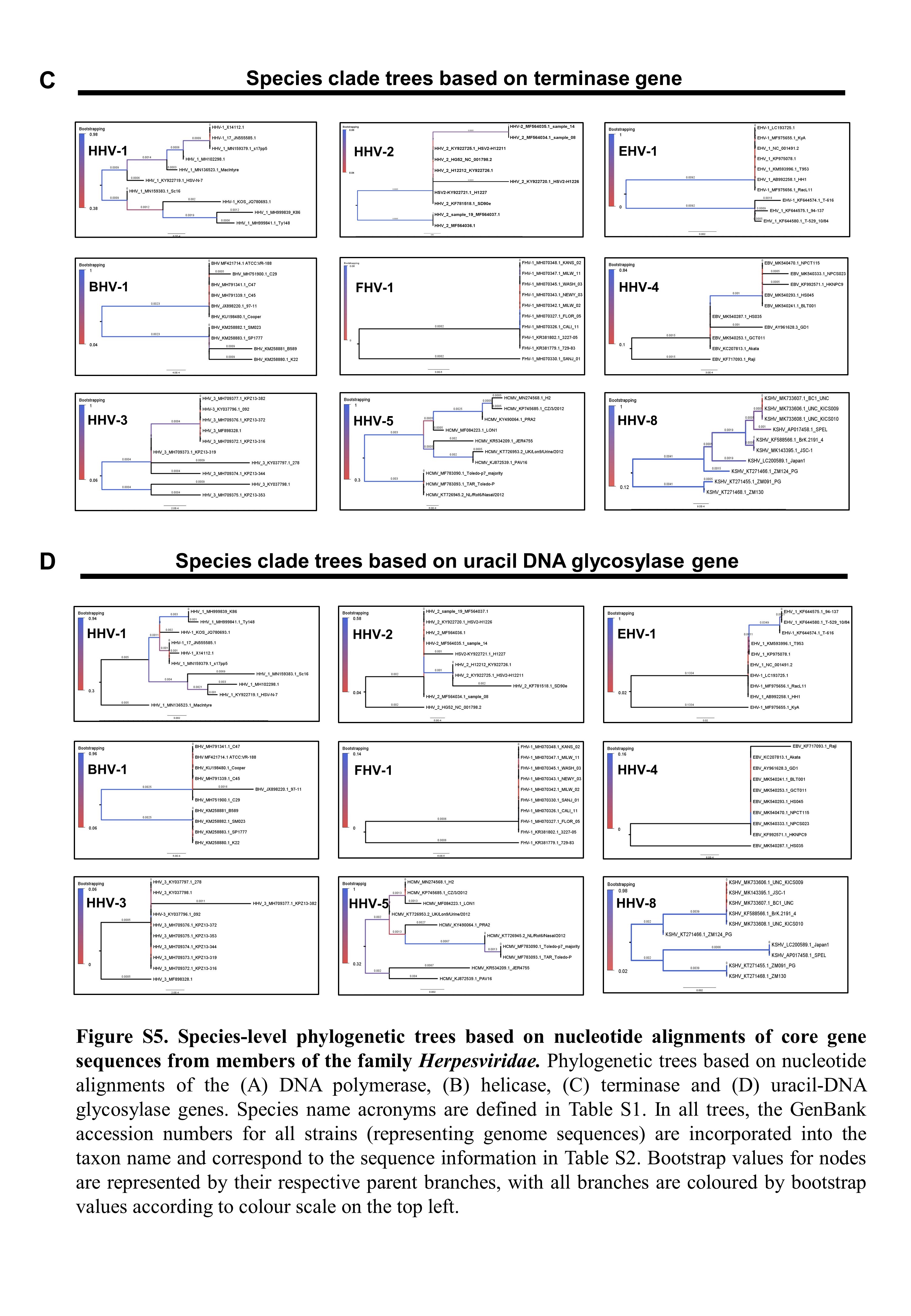

Supplement: Supplementary file 1 [file microorganisms-09-00998-s001.zip › Supplementary Figures S5 FINAL Part 2.JPG]
